# Role of the PhoP–PhoQ gene regulatory system in adaptation of *Yersinia pestis* to environmental stress in the flea digestive tract

**DOI:** 10.1099/mic.0.000082

**Published:** 2015-06

**Authors:** Viveka Vadyvaloo, Austin K. Viall, Clayton O. Jarrett, Angela K. Hinz, Daniel E. Sturdevant, B. Joseph Hinnebusch

**Affiliations:** ^1^​ Paul G. Allen School for Global Animal Health, Washington State University, Pullman, WA, 99164, USA; ^2^​ Plague Section, Laboratory of Zoonotic Pathogens, Rocky Mountain Laboratories, National Institute of Allergy and Infectious Diseases, National Institutes of Health, Hamilton, MT, 59840, USA; ^3^​ Genomics Unit, Research Technologies Section, Rocky Mountain Laboratories, National Institute of Allergy and Infectious Diseases, National Institutes of Health, Hamilton, MT, 59840, USA

## Abstract

The *Yersinia pestis* PhoPQ gene regulatory system is induced during infection of the flea digestive tract and is required to produce adherent biofilm in the foregut, which greatly enhances bacterial transmission during a flea bite. To understand the *in vivo* context of PhoPQ induction and to determine PhoP-regulated targets in the flea, we undertook whole-genome comparative transcriptional profiling of *Y. pestis* WT and Δ*phoP* strains isolated from infected fleas and from temperature-matched *in vitro* planktonic and flow-cell biofilm cultures. In the absence of PhoP regulation, the gene expression program indicated that the bacteria experienced diverse physiological stresses and were in a metabolically less active state. Multiple stress response genes, including several toxin–antitoxin loci and YhcN family genes responsible for increased acid tolerance, were upregulated in the *phoP* mutant during flea infection. The data implied that PhoPQ was induced by low pH in the flea gut, and that PhoP modulated physiological adaptation to acid and other stresses encountered during infection of the flea. This adaptive response, together with PhoP-dependent modification of the bacterial outer surface that includes repression of pH 6 antigen fimbriae, supports stable biofilm development in the flea foregut.

## Introduction

*Yersinia pestis* is the Gram-negative bacterial agent of plague – a rodent-associated flea-borne zoonotic disease that is characterized by epizootic and quiescent inter-epizootic cycles. Within the flea host, the bacteria are able to form a biofilm in the foregut of the digestive tract, which interferes with and can eventually block the ingestion of blood. Complete or partial blockage of the foregut by the biofilm leads to regurgitative transmission of bacteria to the rodent host when the flea attempts to feed (Hinnebusch & Erickson, 2008).

To understand the molecular mechanisms that facilitate biofilm blockage and *Y. pestis* adaptation to the flea gut, we previously compared the transcriptional profiles of *Y. pestis* in blocked fleas and in temperature-matched *in vitro* biofilm and planktonic cultures. One highly transcribed gene that was specifically upregulated in the flea gut encoded PhoP – the transcriptional regulator of the two-component signal transduction system PhoP–PhoQ (Vadyvaloo *et al.*, 2010). The PhoPQ regulatory system is present in a diverse group of Gram-negative bacteria, including both plant and animal pathogens. In general, the PhoPQ system responds to environmental stresses that compromise outer membrane integrity, and in the enteric pathogens many PhoP-regulated genes are involved in virulence and survival within host environments (Groisman, 2001). Mechanistically, PhoQ serves as the sensor histidine kinase in the inner membrane that becomes activated under environmental conditions of low extracellular Mg^2+^ concentration (García Véscovi *et al.*, 1996), mildly acidic pH (Foster & Hall, 1990; Prost *et al.*, 2007) or the presence of cationic antimicrobial peptides (CAMPs) (Bader *et al.*, 2005) and in turn phosphorylates PhoP. Once phosphorylated, PhoP is able to directly or indirectly activate or repress target genes required for adaptation of the organism to its environment.

*In vitro* studies of PhoP homologues of 10 members of the family *Enterobacteriaceae*, including *Y. pestis*, have determined that the PhoP regulon comprises a conserved set of genes, termed the ancestral core genes, as well as a species-specific set of genes (Perez & Groisman, 2009; Perez *et al.*, 2009). Amongst enteric bacteria, the species-specific PhoP regulon has evolved to accommodate individual niche adaptation and modulation of virulence mechanisms (Perez & Groisman, 2009). Direct regulation of transcription depends on PhoP binding to the promoter region, but these genes include other regulatory system components, producing a species-specific cascade effect. For example, only ∼55 *Y. pestis* operons encompassing ∼75 genes have predicted or documented PhoP-binding sites, but PhoP activation by low-Mg^2+^ stress *in vitro* significantly altered the expression of >700 genes (Zhou *et al.*, 2005). In addition, the type of activating stress (low Mg^2+^, acid, CAMPs) can result in different transcriptional responses (Choi *et al.*, 2009; Miyashiro & Goulian, 2007).

In *Y. pestis*, the PhoPQ system greatly enhances intracellular survival in primary phagocytes (Grabenstein *et al.*, 2006; O'Loughlin *et al.*, 2010; Oyston *et al.*, 2000) and resistance to CAMPs (Rebeil *et al.*, 2004, 2013). However, despite the role of PhoP in resisting these innate immune mechanisms, *phoP* mutation in *Y. pestis* has little effect on plague pathogenesis in mice (Bozue *et al.*, 2011; Oyston *et al.*, 2000). In contrast, in keeping with its induction in the flea host, PhoP is required to produce a normal transmissible infection. A *phoP* mutant forms a less cohesive, more fragmented biofilm and has a greatly diminished ability to block the foregut of the flea (Rebeil *et al.*, 2013). The *phoP* mutant is, however, still able to produce a chronic infection in the flea, achieving bacterial loads and infection rates not significantly different from WT. The Mg^2+^ concentration in the flea gut is not limiting, but the pH is mildly acidic and the presence of *Y. pestis* in the blood meal may stimulate the production of CAMPs (Perry & Fetherston, 1997; Rebeil *et al.*, 2013), and these factors might account for the induction of PhoPQ.

The primary aim of this study was to identify the set of PhoP-regulated genes induced or repressed in the flea in order to elucidate genetic mechanisms underlying the role of PhoP in adaptation to the flea gut environment and in the development of the biofilm-mediated blockage that is important for transmission. Following our previous success at obtaining a reproducible and discrete *in vivo* transcriptional profile of WT *Y. pestis* in blocked fleas (Vadyvaloo *et al.*, 2010), we compared the transcriptional profiles of WT and Δ*phoP* *Y. pestis* in the flea gut and in *in vitro* planktonic and biofilm culture conditions. The transcriptome of the *phoP* mutant in the flea and the effect of PhoPQ induction on adaptation to the flea gut are detailed here.

## Methods

### Bacterial strains and growth conditions for *in vitro* transcriptome analyses

The bacterial strains used in this study were *Y. pestis* KIM6+, which lacks the 70 kb virulence plasmid that is not required for flea infection or blockage (Hinnebusch *et al.*, 1996), and an isogenic mutant deleted of functional *phoP* (Rebeil *et al.*, 2013). For *in vitro* planktonic samples, bacteria were grown from frozen stocks in brain heart infusion (BHI) broth at 28 °C, followed by two successive transfers in Luria–Bertani broth supplemented with 100 mM MOPS, pH 7.4 (LB/MOPS) at 21 °C. An inoculum of 10^4^ cells ml^− 1^ was added to 50 ml LB/MOPS and incubated at 21 °C with shaking at 250 r.p.m. until exponential (OD_600_ 2.5) or stationary phases (OD_600_ 4.5). Approximately 0.5 ml of exponential-phase culture and 0.25 ml of stationary-phase culture were resuspended in 1 and 0.5 ml, respectively, of RNAprotect Bacteria Reagent (Qiagen), incubated for 10 min at room temperature, and centrifuged at 21 °C for 5 min prior to RNA extraction.

For *in vitro* biofilms, 400 μl of 10^7^ c.f.u. ml^− 1^ bacterial suspension was injected into a flow cell (Stovall) that was connected to a reservoir of LB/MOPS at 21 °C. Following a 30 min incubation period to allow the bacteria to adhere to the glass surface of the flow cell, LB/MOPS was pumped through the flow cell at a rate of 0.3 ml min^− 1^. After 48 h when the biofilm was fully developed, the flow cell was disconnected and the *Y. pestis* biofilm was harvested and treated with 0.5 ml of RNAprotect similarly to the planktonic cultures. Five independent biological replicates were generated for each condition.

### Construction of *Y. pestis* mutants

Mutants in the *Y. pestis* KIM6+ background were constructed by in-frame deletion of the target gene using one-step inactivation by homologous recombination as described previously (Datsenko & Wanner, 2000; Derbise *et al.*, 2003). The mutant strains were complemented by electroporation of WT promoter and target gene on the pCR4 plasmid (Life Technologies). Primers used for mutant and complement construction are tabulated in Table S7 (available in the online Supplementary Material).

### Flea infection and collection of samples for *in vivo* transcriptome analyses

*Xenopsylla cheopis* fleas were infected with *Y. pestis* KIM6+ and the Δ*phoP* mutant using a previously described artificial feeding system (Hinnebusch *et al.*, 1996). The infectious blood meal was prepared by growing the bacteria at 37 °C in BHI without aeration. A cell pellet containing ∼10^9^ bacteria was resuspended in 1 ml of PBS and this was added to 5 ml of heparinized mouse blood. The infected blood was added to the water-jacketed feeding chamber, which was maintained at 37 °C. The fleas were allowed to feed for 60–90 min through the mouse skin secured over the chamber. Fleas that took a blood meal were maintained at 21 °C and 75 % relative humidity, and fed twice weekly on uninfected mice (Hinnebusch *et al.*, 1996). After 2 weeks, the bacterial numbers in the midgut plateaued, and the incidence of blockage was maximal (Hinnebusch *et al.*, 1996, 2002). At this time point, three independent biological replicates of pooled midguts dissected from ∼30 randomly selected infected fleas were macerated in RNAprotect. Animal use was approved by the Institutional Animal Care and Use Committees (Washington State University and National Institute of Allergy and Infectious Diseases/National Institutes of Health) and was conducted in accordance with institutional guidelines based on the National Institutes of Health Guide for the Care and Use of Laboratory Animals.

### RNA isolation, amplification and microarray

RNA isolation and amplification were carried out as described previously (Vadyvaloo *et al.*, 2010). RNA was isolated from five independent samples from *in vitro* and flow cell cultures, and from three independent biological replicates of pooled flea midguts 2 weeks post-infection, using an RNeasy Mini kit (Qiagen). For a total of five replicates for flea midgut RNA samples, two of the biologically independent RNA extracts were processed in duplicate for microarray analysis. RNA integrity was verified on a Bioanalyzer 2100 (Agilent Technologies). Total RNA (100 ng) was amplified and labelled with biotin-16-UTP (Roche Molecular Biochemicals) by using a Message-Amp II-Bacteria Amplified antisense RNA kit (Ambion). Amplified RNA was then fragmented using Ambion's Fragmentation reagent (Applied Biosystems), hybridized to the RML custom Affymetrix GeneChip that contains sequences for all *Y. pestis* predicted ORFs and scanned.

### Microarray data analysis

Affymetrix GeneChip Operating Software version 1.4 (http://www.affymetrix.com) was used for initial analysis of the microarray data at the probe-set level. All *.cel files, representing individual biological replicates, were scaled to a trimmed mean of 500 using a scale mask consisting of only the *Y. pestis* KIM6+ probe sets to produce the *.chp files. A pivot table with all samples was created including calls, call *P* value and signal intensities for each gene. The pivot table was then imported into GeneSpring GX 7.3 (http://www.chem.agilent.com), where hierarchical clustering (condition tree) using a Pearson correlation similarity measure with mean linkage was used to produce the dendrogram indicating that biological replicates grouped together. The pivot table was also imported into Partek software (Partek) to produce a principal components analysis plot as a second statistical test for the grouping of biological replicates. An ANOVA was performed on this dataset to produce *P* values and these *P* values were multiple-test corrected using a Benjamini–Hochberg false-discovery rate method for each comparison of interest.

The means of the replicates of all test conditions and controls were combined, with differential expression determined by using the multiple-test corrected *P* value significance level of 0.05 and a fold-change Ţ2 or 0.001 and a fold-change Ţ1.5. The microarray data can be downloaded from the Gene Expression Omnibus public database (accession number GSE61558).

### Reverse transcription-quantitative PCR (RT-qPCR)

Bacterial cells pelleted following treatment with RNAprotect were resuspended in 100 μl of 2 mg ml^− 1^ lysozyme solution and incubated for 10 min at room temperature. RNA was isolated using an RNeasy Plus (Qiagen) kit as per the manufacturer's instructions, except that the bacterial lysate was aspirated through a 22 gauge needle once RLT buffer had been added. Total RNA samples were then treated with DNase I (Ambion) for 1 h and concentrated using an RNeasy MinElute Cleanup kit (Qiagen). RNA concentration was determined by using a NanoDrop spectrophotometer (Thermo Scientific) and RNA integrity verified by an Agilent Bioanalyzer. Approximately 10 μg of total RNA was converted into cDNA using random hexamers and SuperScript III (Life Technologies) as per the manufacturer's instructions.

Samples were amplified using oligonucleotide primers for *crr* (y1485), y1667, y0666, y3909, y3519, y2882 (*psaA*), y1918 and the IQ SYBR Green Supermix (Bio-Rad) via a two-step protocol on a Bio-Rad CFX384 real-time system. Oligonucleotide sequences are tabulated in Table S1. Cycling parameters were optimized using *Y. pestis* KIM6+ genomic DNA. Expression levels of the genes of interest were determined as a ratio to the levels of the constitutively expressed gene *crr* (Vadyvaloo *et al.*, 2010).

### Acid tolerance assay by *A*_460_ measurement of formazan accumulation

Bacterial cultures were inoculated from − 80 °C glycerol stocks into BHI medium with the appropriate antibiotic and grown overnight at 28 °C. The bacteria were then subcultured overnight in LB medium and diluted 1 : 10 in LB medium, and incubation was continued at 28 °C. Following ∼5 h incubation (*A*_600_∼0.5), 200 μl of culture was transferred to 2 ml of 1 ×  PBS and to 2 ml of LB, pH 4.5, and incubated for 10 min. Metabolic viability was quantified using a Dojindo Microbial Viability kit (Dojindo Molecular Technologies) as a measure of bacterial survival. At this point, an aliquot of the WT strain samples was treated with RNAprotect to prepare for RT-qPCR analysis. Experiments were performed in triplicate, and one-way ANOVA with Tukey's post-test were used to determine significance.

### Autoaggregation assays in acidic medium

Bacterial cultures were inoculated from − 80 °C glycerol stocks into BHI medium with the appropriate antibiotic and grown overnight at 28 °C. The bacteria were then subcultured to LB supplemented with 4 mM MgCl_2_ and 4 mM CaCl_2_, pH 7.0, and allowed to grow overnight. This culture was used to inoculate glass Kimax tubes of LB/4 mM MgCl_2_/4 mM CaCl_2_, pH 5.5 or 7.0, and cultures were allowed to grow overnight with shaking at room temperature. A visual inspection of biofilm formation was made; then the cultures were vortexed to resuspend the cells and the OD_600_ was recorded. The cultures were allowed to sit for a further 24 h, after which the OD_600_ was recorded a final time to determine the percentage sedimentation. The percentage sedimentation was calculated by dividing the final OD_600_ by the OD_600_ taken immediately after vortexing and subtracting this percentage from 100%. One-way ANOVA with Tukey's post-test was used to determine significance.

## Results

### Transcriptional profile of WT versus Δ*phoP* *Y. pestis*

To identify genes regulated by PhoP during flea infection, we infected fleas with either WT or Δ*phoP* *Y. pestis* strains, and bacteria recovered from dissected flea digestive tracts 2 weeks after infection were prepared for microarray analysis. At this time point, although the incidence of foregut blockage was approximately three times lower in the Δ*phoP*- than in the WT-infected fleas, the mean bacterial load was >10^5^ bacteria per flea for both strains (Rebeil *et al.*, 2013). Transcriptome analyses of the two strains from flow-cell biofilms and from exponential- and stationary-phase planktonic cultures grown at 21 °C, the same temperature at which the fleas were maintained, were also performed.

**Fig. 1.**
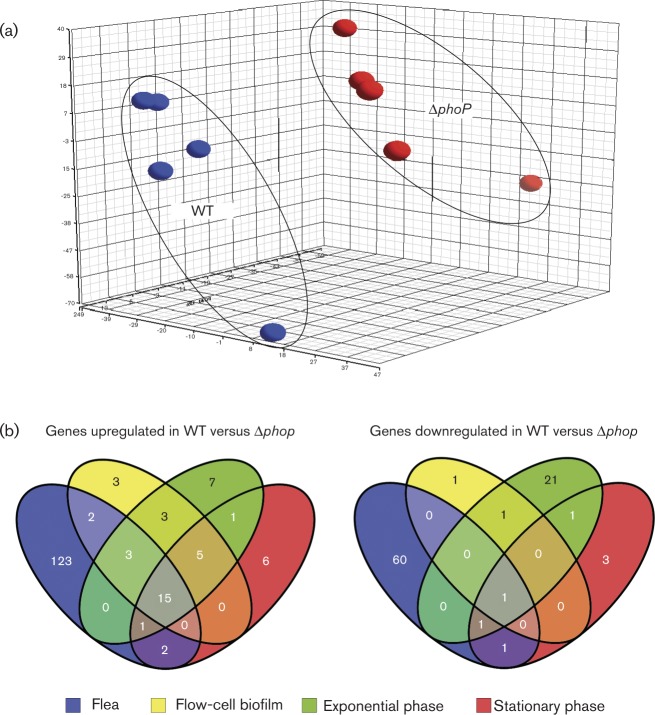
Distinct transcriptional profile of the *Y. pestis* Δ*phoP* mutant in the flea. (a) Principal components analysis of five independent replicate transcriptional profiles of *Y. pestis* WT and isogenic Δ*phoP* strains during flea gut infection. (b) Venn diagrams representing the number of genes significantly upregulated (left) or downregulated (right) in WT *Y. pestis* relative to the Δ*phoP* mutant under different environmental conditions.

In the flea, significant expression of 49 and 44 % of *Y. pestis* ORFs was detected in the WT and Δ*phoP* strains, respectively. In stationary- and exponential-phase planktonic and in flow-cell biofilm conditions, expression of ∼75 % of *Y. pestis* ORFs was detected. Principal component analysis, used to represent the overall variance of the data, indicated distinct and reproducible gene expression profiles for both the Δ*phoP* mutant and the WT strain during infection of the flea (Fig. 1a) and *in vitro* conditions (Fig. S1). Validation of the microarray data was achieved with RT-qPCR, which demonstrated consistent gene expression changes for several genes using both methods (Fig. S2). In addition, transcriptional changes of several genes expressed in the flea relative to *in vitro* conditions from a previous transcriptional study (Vadyvaloo *et al.*, 2010) of *Y. pestis* in fleas were compared with these data (Fig. S3), revealing consistent gene expression differences. A total of 146, 31, 35 and 30 genes were expressed significantly more highly by WT *Y. pestis* in the flea, *in vitro* biofilm, and exponential- and stationary-phase planktonic growth conditions, respectively (Fig. 1b, Tables S1, S2A, S3A and S4A). Conversely, 63, three, 25 and seven genes were expressed significantly more highly by the *phoP* mutant under the same conditions (Fig. 1b, Tables S2, S3B, S4B, and S5B). The cumulative number of genes differentially expressed in the flea accounted for 9 % of the total transcribed *Y. pestis* ORFs. As expected, as LB did not contain any known inducers of PhoPQ, only ∼1 % of the total transcribed ORFs were differentially expressed in the *in vitro* culture conditions (Fig. 1b). Thus, induction of the PhoPQ system had a marked effect on the transcriptome of *Y. pestis* in the flea.

### Subsets of *Y. pestis* genes regulated similarly by PhoP in more than one environmental condition

Previous *in vitro* gene expression and PhoP-binding studies employing low-Mg^2+^ PhoPQ-inducing conditions combined with bioinformatic approaches delineated a PhoP-regulated gene repertoire in *Y. pestis* (Harari *et al.*, 2010; Li *et al.*, 2008; Perez *et al.*, 2009; Zhou *et al.*, 2005). Here, our transcriptomic comparisons (Fig. 1b) revealed a common or conserved set of PhoP-regulated genes irrespective of growth environment, as well as other genes that were differentially regulated in the flea and *in vitro* biofilms only or in planktonic cultures only (Table 1). The genes identified here as being differentially regulated under all conditions were previously determined to be directly regulated by PhoP *in vitro*, except for the putative glycosyltransferase gene y3968 (Harari *et al.*, 2010; Li *et al.*, 2008; Perez & Groisman, 2009; Perez *et al.*, 2009; Zhou *et al.*, 2005). Amongst these conserved PhoP-regulated genes were members of the *pbgP*–*pmrF*–*pmrI*–*pmrJ*–*arnT*–*pmrL*–*pmrM* operon and *ugd* that were responsible for modification of the LPS lipid A with aminoarabinose that increases the net positive cell surface charge and confers resistance to CAMPs (Gunn *et al.*, 2000; Rebeil *et al.*, 2013; Winfield *et al.*, 2005). Interestingly, *pagP*, a PhoP-regulated gene which acts to protect against CAMPs via acylation of lipid A (Guo *et al.*, 1998; Harari *et al.*, 2010), was not induced in the flea, but was highly induced *in vitro* (Table 1).

**Table 1:** Genes with a shared PhoP-dependent differential expression pattern in more than one environmental condition Criteria for significance: ≥ 2-fold difference, *P* ≤ 0.05 or ≥ 1.5-fold difference, *P* ≤ 0.001. Fold-change values in parentheses not significant based on *P* value. Genes in bold type are associated with an upstream consensus PhoP-binding site and are predicted or have been shown to be directly regulated by PhoP (Harari *et al.*, 2010; Li *et al.*, 2008; Perez *et al.*, 2009).

			Relative expression (fold-change; WT versus Δ*phoP*) during growth in			
Gene ID	Gene name	Predicted function of gene product	Flea	LB (flow cell)	LB (exponential)	LB (stationary)
**Significantly higher expression in WT *Y. pestis* in all conditions**						
**y1795**	–	Predicted outer membrane protein; next to *phoP*	7.2	20.7	57.5	18.5
**y1877**	–	Predicted *N*-acetylmuramoyl-l-alanine amidase	2.5	7.2	13.8	4.7
**y1917** ^*^	***pgbP*** (***pmrH***)	Lipid A modification; aminoarabinose synthesis	2.8	16.2	18.2	8.6
**y1918** ^*^	***pmrF***	Lipid A modification; UDP-aminoarabinose transferase	5.2	11.6	16.7	7.4
**y1919** ^*^	***pmrI***	Lipid A modification; UDP-glucuronic acid decarboxylase/ UDP-aminoarabinose formyltransferase	3.0	10.7	17.1	8.8
**y1920** ^*^	***pmrJ***	Lipid A modification; UDP-aminoarabinose deformylase	3.0	13.3	14.9	6.7
**y1921** ^*^	***pmrK*** **(*arnT*)**	Lipid A modification; aminoarabinose transfer	1.9	7.8	13.9	4.7
**y1922** ^*^	***pmrL***	Lipid A modification; aminoarabinose phospho-UDP flippase subunit	1.6	6.6	8.8	3.1
**y1923** ^*^	***pmrM***	Lipid A modification; aminoarabinose phospho-UDP flippase subunit	2.2	11.5	12.8	4.1
**y2124**	–	Putative inner membrane protein	5.1	16.0	31.7	11.2
**y2147**	***ugd***	Lipid A modification; aminoarabinose synthesis	4.5	7.6	15.1	4.7
**y2608**	–	Predicted nucleoside-diphosphate sugar epimerase	3.3	15.4	24.0	15.2
**y2859**	–	Undecaprenyl-pyrophosphate phosphatase; *ybjG* homologue	3.2	6.8	9.5	4.6
y3968	–	Putative glycosyltransferase	2.8	3.1	3.5	3.1
YPO1659^+^ PhoP binding†	–	Hypothetical protein; next to *mgtC*	3.6	17.4	27.7	15.9
**Significantly higher expression in Δ*phoPY. pestis* in all conditions**						
y2882	*psaA*	pH 6 antigen fimbrial subunit	− 81.7	− 26.3	− 61.0	− 130.4
**Significantly higher expression in *WTY. pestis* in biofilm conditions (flea and flow cell) only**						
y1741	*rcsA*	Transcriptional regulator RcsA; Rcs regulatory system (pseudogene)	4.9	2.8	(2.2)	(2.0)
**y1792**	–	Hypothetical protein; next to *phoQ*	2.1	2.0	(1.7)	1.1
**Significantly higher expression in *WTY. pestis* during *in vitro* growth only**						
**y0447**	–	Membrane-associated alkaline phosphatase	(1.5)	4.0	4.0	4.0
**y1820**	***mgtC***	Mg^2+^ transport ATPase protein C	1.3	12.5	19.4	6.1
**y2563**	***pagP***	Lipid A acylation; palmitoyltransferase	1.1	20.4	46.6	9.9
**y2816**	–	*virK*-like virulence factor	(1.5)	17.4	28.5	24.9
**y2858**	–	Membrane protein	(1.9)	5.4	7.0	2.9

* Linked genes.

† Gene not annotated in *Y. pestis* KIM (YPO number indicates *Y. pestis* CO92 homologue).

The *psaA* gene was very highly repressed in the WT strain relative to the *phoP* mutant under all conditions. PsaA is the structural subunit of the pH 6 antigen fimbriae, an antiphagocytic virulence factor produced at 37 °C (Lindler & Tall, 1993; Lindler *et al.*, 1990). Other genes in the pH 6 antigen locus, including the transcriptional activator *psaE*, were significantly upregulated in the *phoP* mutant, both in the flea and in planktonic cultures (Tables S2–S6). PhoP has been shown to bind to the proximal promoter region of both the *psaABC* pH 6 operon and its associated regulatory *psaEF* operon, and to repress their expression (Zhang *et al.*, 2013), whereas the RovA transcriptional regulator activates *psa* gene expression (Cathelyn *et al.*, 2006). Thus, the previously described repression of *rovA* and induction of *phoP* in fleas (Vadyvaloo *et al.*, 2010) likely both contribute to the absence of *psa* expression in WT *Y. pestis* during flea infection. Furthermore, PhoP negatively regulates *rovA* (Zhang *et al.*, 2011) and *rovA* was expressed 1.6-fold higher by the *phoP* mutant in the flea. Therefore, loss of PhoP would be predicted to release repression of the *psa* genes both by the absence of the repressor (PhoP) and indirectly by derepression of the activator (RovA), resulting in the extremely high relative expression of the *psa* genes in the *phoP* mutant.

Two genes were differentially expressed only in biofilm growth conditions (Table 1). One was *rcsA*, a component of the Rcs gene regulatory system, which acts to repress biofilm growth in *Yersinia* (Sun *et al*., 2012). However, *rcsA* is a pseudogene in *Y. pestis*, and loss of function of this gene was an essential step in the evolution of flea-borne transmission because it significantly enhanced biofilm formation (Sun *et al.*, 2008, 2014).

### *Y. pestis* genes specifically upregulated by PhoP only in the flea gut environment

The majority of the genes (123 of 146) that were expressed significantly more highly in fleas infected with WT than with Δ*phoP* *Y. pestis* were not differentially regulated in any of the *in vitro* growth conditions (Fig. 1b, Table S2). These genes included, notably, *phoQ*, four genes in the *lsr* locus, and the *yit* and *yip* insecticidal-like toxin loci. The *lsr* genes are responsible for the synthesis and transport of autoinducer-2 and comprise one of the three *Y. pestis* quorum-sensing systems (Yu *et al.*, 2013). Quorum sensing is associated with biofilm growth; however, a *Y. pestis* mutant lacking all three quorum-sensing systems did not have a notable defect in flea infection and blockage phenotypes (Jarrett *et al.*, 2004). Thus, upregulation of the *lsr* genes may be a consequence rather than a cause of the more dense biofilm produced in the flea by WT *Y. pestis* compared with the *phoP* mutant, although autoinducer-2 quorum sensing is also associated with certain metabolic activities and the oxidative stress response (Yu *et al.*, 2013). Genes in the *yit* and *yip* insecticidal-like toxin loci are amongst the most highly expressed in the flea (Vadyvaloo *et al.*, 2010), and there is evidence that they are PhoP-regulated (Harari *et al.*, 2010); however, like the quorum-sensing systems, the *yit/yip* locus is not required to produce normal infection or biofilm-dependent blockage rates in the flea (Spinner *et al.*, 2012).

Our previous microarray study indicated that amino acids, particularly the glutamate family, are the predominant carbon and energy sources utilized by *Y. pestis* in the flea (Vadyvaloo *et al.*, 2010). Of the 146 genes that were expressed significantly higher by WT *Y. pestis* in the flea, 33 were involved in amino acid transport and metabolism, and many others were involved in other basic metabolic functions (Table S2). The decreased transcription of genes encoding metabolic processes by the *phoP* mutant in the flea gut was not compensated by a notable induction of genes encoding alternate metabolic pathways, indicating that the *phoP* mutant was much less metabolically active in the flea gut than WT *Y. pestis*. These results indicated that normal *Y. pestis* metabolism in the flea gut was dependent on induction of PhoPQ.

Four genes in the *nhaC* locus (y3550, y3553–y3555) previously characterized as amongst the most highly upregulated genes in the flea (Hinnebusch *et al.*, 2012; Vadyvaloo *et al.*, 2010) were comparatively downregulated in the *phoP* mutant (Table S2). NhaC is an Na^+^/H^+^ antiporter whose expression is PhoP-regulated (Harari *et al.*, 2010). The transcriptional activator of *nhaA*, which encodes a second Na^+^/H^+^ antiporter, was also more highly expressed by WT *Y. pestis* in the flea (4.7-fold difference; Table S2).

### Genes encoding several general stress adaptation mechanisms are induced in the *Y. pestis* *phoP* mutant during flea gut infection

Of the 63 genes that were more highly expressed by the *phoP* mutant than by WT *Y. pestis* in the flea, 60 were specifically upregulated only in the flea gut and not under any other growth conditions (Table S6, Fig. 1b). Amongst these were several genes that encoded stress response functions, including heat-shock proteins and the heat-shock sigma factor *rpoH* (Table 2). The small RpoH-regulated heat-shock protein genes *ibpA* and *ibpB*, which were directly repressed by PhoP *in vitro* (Perez *et al.*, 2009), were also much more highly expressed in the *phoP* mutant. In addition to the general heat-shock stress response, *cpxP*, a component of the Cpx envelope stress system, was highly upregulated during flea infection in the *phoP* mutant. The *terZABCDE* tellurite resistance operon, so-named because it confers protection against antibacterial tellurium compounds, was also induced in the absence of PhoP in the flea. The *ter* genes encode a stress response system that also controls resistance to pore-forming colicins and bacteriophage (Whelan *et al.*, 1995). In *Y. pestis*, the *ter* operon is induced by polymyxin B (a CAMP) and other antibiotics, and is directly activated by the oxidative stress response regulator OxyR (Ni *et al.*, 2014; Zhou *et al.*, 2006).

**Table 2:** Stress response genes upregulated in *Y. pestis* Δ*phoP* during flea infection Criteria for significance: ≥ 2-fold difference, *P* ≤ 0.05 or ≥ 1.5-fold difference, *P* ≤ 0.001. Fold-change values in parentheses not significant based on *P* value. Genes in bold type are associated with an upstream consensus PhoP-binding site and are predicted or have been shown to be directly regulated by PhoP (Harari *et al.*, 2010; Li *et al.*, 2008; Perez *et al.*, 2009).

			Relative expression (fold-change; WT versus Δ*phoP*) during growth in:			
Gene ID	Gene name	Predicted function of gene product	Flea	LB (flow cell)	LB (exponential)	LB (stationary)
**Envelope stress/general stress response**						
y0066	*cpxP*	Periplasmic stress adaptor protein	− 5.5	( − 2.0)	( − 2.0)	4.5
y0137	*degQ*	Endopeptidase; HtrA family protein quality control and stress response	− 1.7	1.2	− 1.2	1.0
y0295	*hslV*	Endopeptidase; protein quality control	− 2.1	− 1.2	− 1.2	1.0
y0419	*rpoH*	RNA polymerase factor sigma-32	− 2.1	− 1.1	− 1.1	− 1.3
y1265	*kdpA*	Potassium-transport, osmotic stress	− 2.1	1.2	− 1.0	− 1.3
y1266	*kdpB*	Potassium-transport, osmotic stress	− 3.1	1.1	− 1.2	(1.5)
y1868	*htpX*	Heat-shock protein	− 2.8	− 1.1	− 1.3	1.3
y3706	*dnaK*	Heat-shock protein	− 3.0	− 1.3	1.1	1.2
**y4101**	***ibpA***	Heat-shock protein chaperone	− 5.2	1.3	1.2	1.3
**y4102**	***ibpB***	Heat-shock protein	− 3.9	(1.6)	− 1.1	1.4
**Tellurite resistance genes**						
y0555	*terZ*	Tellurite resistance, stress response protein	− 3.7	− 1.3	− 1.2	− 1.4
y0556	*terA*	Tellurite resistance, stress response protein	− 2.5	1.0	1.0	1.4
y0557	*terB*	Tellurite resistance, stress response protein	− 2.2	1.2	− 1.2	1.0
y0558	*terC*	Tellurite resistance, stress response protein	− 2.4	1.0	− 1.1	1.1
**TA**						
Y1074	–	*relE/parE* family addiction toxin module	− 3.8	( − 1.7)	( − 2.0)	1.0
Y1075	–	*phD* family addiction antitoxin module	− 2.5	( − 1.6)	( − 1.8)	1.0
y3266	*mqsA*	MqsA antitoxin	− 3.5	( − 1.8)	− 1.4	1.0
YPO0882^*^	*mqsR*	MqsR toxin	− 4.5	( − 2.6)	− 1.4	1.0
YPO1087^*^	–	HigB2 toxin	− 2.3	− 1.2	1.0	1.1
**YhcN family (DUF1471) multiple stress resistance**						
**y0666**	***yhcN***	Multiple stress resistance protein	− 9.3	( − 1.5)	− 1.3	1.1
y1667	–	Multiple stress resistance protein	− 12.4	( − 1.9)	− 1.0	− 1.2

* Gene not annotated in *Y. pestis* KIM (YPO number indicates *Y. pestis* CO92 homologue).

Elevated transcription of four putative toxin–antitoxin (TA) loci (Goulard *et al.*, 2010) was observed in the *phoP* mutant during flea infection, but not during *in vitro* biofilm or planktonic growth (Table 2). TA loci may enhance bacterial survival as their transcription is stimulated when the cells encounter nutritional or other environmental stress (Christensen-Dalsgaard *et al.*, 2010; Christensen *et al.*, 2001; Gerdes *et al.*, 2005; Ramage *et al.*, 2009). Subsequent toxin accumulation inhibits essential energy-requiring cellular processes, such as replication and translation, leading to a reversible bacteriostasis that enables the cells to survive stressful periods (Buts *et al.*, 2005; Gerdes *et al.*, 2005).

### Genes encoding YhcN family proteins function as acid stress adaptation factors in *Y. pestis*

Three of the most highly upregulated genes (y0666, y1667 and y3519) in the *phoP* mutant (Tables 2 and S6) were homologous to genes induced in *Escherichia coli* biofilms (Hancock & Klemm, 2007; Hancock *et al.*, 2010; Zhang *et al.*, 2007). The y3519 gene (expressed 13.1-fold higher by the *phoP* mutant relative to WT *Y. pestis* in the flea) was predicted to encode an extradiol-dioxygenase of unknown function. The y0666 (9.3-fold increase) and y1667 (12.4-fold increase) genes contained a domain of unknown function designated DUF1471 and encoded YhcN family proteins – a conserved group of low-molecular-mass secreted or periplasmic proteins (Rudd *et al.*, 1998). The *Y. pestis* KIM6+ genome contained five YhcN protein family genes (y1667, y0666, y0640, y3909 and y2136) that encoded predicted proteins of 86, 87, 53, 92 and 317 amino acids, respectively. In *E. coli*, four of the 10 YhcN family proteins were shown to influence biofilm formation and at least two participated in the general stress response, conferring increased resistance to acid, heat, oxidative and heavy metal stresses (Lee *et al.*, 2010; Weber *et al.*, 2010; Zhang *et al.*, 2007). The y1667 and y3519 genes were also significantly more highly expressed by WT *Y. pestis* in the flea than in LB *in vitro* planktonic or biofilm cultures (Vadyvaloo *et al.*, 2010).

**Fig. 2.**
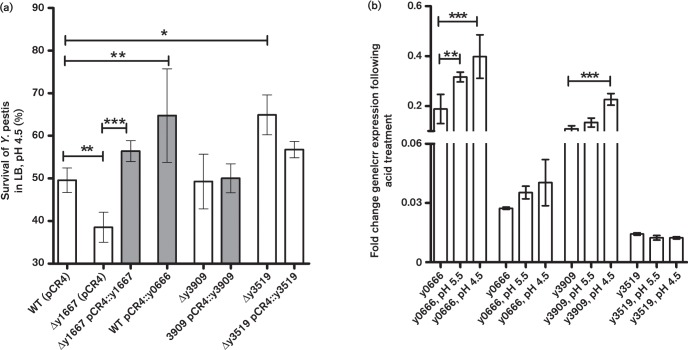
*Y. pestis* YhcN family genes highly induced in the flea gut are involved in an acid stress response. (a) Viability of *Y. pestis* cells assessed by formazan dye accumulation following a 10 min exposure to pH 4.5 and calculated as the ratio of the *A*_460_ of 1 ml aliquots of cells resuspended in LB pH 4.5 and 7.0, respectively. (b) Relative transcription of the y0666, y1667, y3909 and y3519 genes following exposure of *Y. pestis* to acidic pH. The RT-qPCR transcript values were normalized to the constitutively expressed *crr* gene.

Given that the PhoPQ system is induced by acidic conditions that are thought to be characteristic of the flea gut and that PhoPQ induction promotes acid tolerance (Foster & Hall, 1990), it is plausible that other factors that mediate acid tolerance are induced to compensate for the absence of PhoP. To test the role of the YhcN family genes and y3519 in acid tolerance and adaptation in *Y. pestis*, we generated mutant strains in which the y1667, y3909 and y3519 genes were deleted. Unable to successfully generate a mutant of the y0666 gene, an opposite strategy in which the WT strain was transformed with a high-copy-number plasmid carrying the y0666 gene and native promoter region (pCR4 :: y0666) was used. Additionally, the y3909 gene was selected to be deleted due to its similar size to y0666 and y1667, to provide proof of principle for the phenotypes exhibited by genes encoding YhcN proteins. The three mutant strains Δy1667, Δy3909 and Δy3519, and the respective complemented strains Δy1667 (pCR4 :: y1667), Δy3909 (pCR4 :: y3909) and Δy3519 (pCR4 :: y3519), were evaluated for their survival response to acid treatment at pH 4.5 (Fig. 2a). Only the y1667 mutant was significantly more susceptible to acid, and transcomplementation restored tolerance to acid pH at slightly higher levels than the WT strain (Fig. 2a). Increased tolerance to low-pH stress was observed in the WT strain transformed with pCR4 :: y0666 compared with the WT strain transformed with the pCR4 plasmid alone. Coupled with this, a significant increase in transcription of y0666 was observed following acid exposure, which correlated with increasing acidity of the medium (Fig. 2b). Based on these findings, a role in acid stress adaptation could be inferred for the YhcN family genes y1667 and y0666. The y3909 gene showed significantly increased transcription at the more acidic pH of 4.5, but deletion of y3909 did not reduce acid tolerance (Fig. 2).

**Fig. 3.**
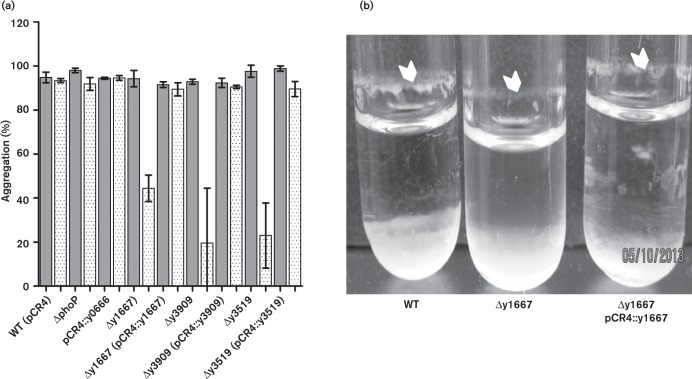
*Y. pestis* genes highly induced in the flea gut are involved in aggregation and biofilm phenotypes. (a) Bacterial autoaggregation was assessed by measuring the sedimentation of bacterial growth in static overnight LB/4 mM MgCl_2_/4 mM CaCl_2_ cultures (pH 5.5 and 7) incubated at room temperature. The mean ± sd of at least three independent replicates is indicated, grey bars denote ph7 and dotted bars denote pH5.5. (b) Image of biofilm formation in LB/4 mM MgCl_2_/4 mM CaCl_2_, pH 5.5 medium following vigorous overnight shaking in glass tubes at room temperature.

An interesting phenomenon of increased aggregation of bacterial cells in low pH was observed (Fig. S4). Therefore, we assessed the contribution of the y1667, y3909 and y3519 genes to aggregation and biofilm formation at mildly acidic pH 5.5. Although their growth rates in LB pH 5.5 were equivalent to WT, the Δy1667, Δy3909 and Δy3519 mutants showed significantly decreased autoaggregation, as measured by sedimentation assay (Fig. 3a). As biofilm detached easily from polystyrene plates and glass tubes in low-pH media, it was not possible to quantify biofilm formation. Instead, the biofilm phenotype was visually documented (Fig. 3b), showing that the Δy1667 mutant formed less biofilm at pH 5.5 compared with the WT and complemented strains. The Δy1667 strain was the only one of the four mutants that lacked the ability to form a thick biofilm at pH 5.5 (data not shown). The Δy1667 mutant, however, displayed no defect in biofilm-dependent blockage or survival rates during flea infection (Table S6). All mutant strains formed WT levels of biofilm in LB pH 7.0 *in vitro* (data not shown).

## Discussion

The life stage of *Y*. *pestis* in the flea is fundamentally characterized by the formation of a bacterial biofilm that facilitates stable colonization of the flea gut and eventual transmission. Shortly after an infectious blood meal, multicellular aggregates of *Y. pestis* enclosed within an extracellular matrix form in the flea digestive tract. Those in the lumen of the midgut can persist for several weeks and cause little or no morbidity to the flea. Those that localize to the foregut, however, eventually impede blood feeding enough to result in regurgitative transmission and death of the flea by starvation. Bacterial biofilm development involves complex genetic pathways and the phenotype is strongly influenced by environmental factors, such as the substrate and surrounding medium. Our understanding of the mechanisms underlying biofilm development in *Y. pestis* is rudimentary. As is generally true for bacteria, synthesis and maintenance of intracellular cyclic-di-GMP leading to the production of an extracellular polysaccharide matrix are minimum requirements (Hinnebusch & Erickson, 2008). *Y. pestis* forms biofilms only at temperatures below ∼26 °C, typical of the flea host, but few details of regulation are known.

The PhoPQ system is not required for *Y. pestis* biofilm formation, but alters the biofilm phenotype. A Δ*phoP* mutant forms a less dense and less cohesive biofilm, both *in vitro* and in the flea (Rebeil *et al.*, 2013; Sun *et al.*, 2009). The PhoPQ system is induced in the flea gut, but the inducing stimulus is unknown. The physiological environment of the flea gut has yet to be defined with respect to its physico-chemical properties (pH, oxygen tension, osmolarity) and its innate immune response to infection. A further limitation in defining this environment has been the lack of a genome sequence for the flea. These limitations have mostly confined understanding of the flea/*Y. pestis* interaction to bacterial-centric views and approaches. Only a single study has examined the flea response to *Y. pestis* infection, which suggested that fleas can generate reactive oxygen species upon infection with *Y. pestis* (Zhou *et al.*, 2012). Any other potential antibacterial factors in the infected flea gut are undefined.

A total of 183 genes were differentially expressed by WT *Y. pestis* relative to the Δ*phoP* mutant in fleas examined 2 weeks after infection, when mature biofilms had formed in the digestive tract. A large proportion of these genes are involved in metabolism and stress response. Most of them are probably not part of the PhoPQ regulon or the biofilm development pathway per se. Instead, their dysregulation likely indicates that the mutant bacteria are subjected to stresses normally alleviated by PhoPQ induction and that they subsequently generate an altered physiological response to this unresolved stress. For example, WT *Y. pestis* cells enclosed within a mature biofilm might be shielded from potential stressors in the flea gut, such as pH, digestive enzymes and CAMPs. The observation of increased aggregation in mildly acidic conditions *in vitro* is conducive to formation of a self-protective cohesive biofilm by *Y. pestis* in the flea gut once it encounters this low-pH environment. The much less dense structure and other physico-chemical properties of the mutant biofilm may allow permeation of flea gut contents, thereby exposing the bacteria to increased and more sustained physiological stress. Along with upregulation of several stress response systems in the absence of PhoP, many metabolism genes were downregulated in the flea compared with WT *Y. pestis*, indicative of a general slowdown in carbon- and energy-requiring cellular processes. Taken together, the transcriptome comparison identified not only the subset of the PhoP regulon specific to induction of the PhoPQ system in the flea, but also other sets of genes, not part of the PhoP regulon, that indicate that Δ*phoP* mutant cells are responding to diverse physiological stresses in the flea gut and are in a metabolically less active state.

Biofilm formation can be a self-protective mechanism in response to suboptimal environmental conditions, and there is some overlap between the transcriptomic responses to various stresses and biofilm formation (Landini, 2009). For example, *E. coli* YhcN family genes, which function to protect against acid and other stresses, are also involved in biofilm formation (Hancock *et al.*, 2010; Weber *et al.*, 2010; Zhang *et al.*, 2007, 2008). We found that *Y. pestis* YhcN family homologues were also induced during mild acid stress, and were associated with increased acid resistance and biofilm formation *in vitro*. The flea digestive tract, although not a low-Mg^2+^ environment, is mildly acidic (Perry & Fetherston, 1997). PhoPQ has been shown to be induced by and to protect against acid stress in *Salmonella* (Bearson *et al.*, 1998; Foster & Hall, 1990), suggesting that low pH is the environmental stimulus for PhoPQ induction in the flea, and that the y1667 and y0666 YhcN family genes are induced in the flea to compensate for the absence of PhoP. Flea CAMPs are a potential alternate stimulus, because the midgut epithelium of other blood-feeding insects is known to produce CAMPs in response to an infected blood meal (Dimopoulos *et al.*, 1997; Lehane *et al.*, 1997). However, *Y. pestis* mutants that are highly susceptible to CAMPs replicate normally in the flea gut, suggesting that oral infection of the flea does not induce inhibitory levels of CAMPs in the digestive tract (Rebeil *et al.*, 2013).

Elevated transcription of four putative TA loci was observed in the *phoP* mutant. Ten chromosomally encoded putative TA modules and two putative solitary antitoxin genes that belong to five separate TA families have been described in *Y. pestis* (Goulard *et al.*, 2010). Under artificially inducible conditions, the *higB2* and *relE1* toxins are toxic to *Y. pestis* growing *in vitro* at 37 °C (Goulard *et al.*, 2010). The homologous *mqrsRA*, *relE1B1* and *higB2A2* TA modules of the *Y. pestis* 91001 strain are induced by high-salt, low-osmolarity and low-Mg^2+^ stress (Han *et al.*, 2005, 2007). In other bacteria, homologues of *relE1* are transcribed during amino acid and glucose starvation (Christensen *et al.*, 2001), whilst *higB2* homologues are induced by numerous stress conditions, such as amino acid starvation, exposure to antibiotics and hypersaline osmotic shock (Han *et al.*, 2005, 2007). One of the upregulated putative TA pairs (Y1074–Y1075) has not previously been described as a TA locus and is encoded on the largest *Y. pestis* plasmid, pMT-1, which is essential for survival of *Y. pestis* in the flea (Hinnebusch *et al.*, 2002). Plasmid-borne TA modules exert their toxic effects in a manner that promotes plasmid stability by destroying plasmid-cured progeny (Hayes, 2003). *In vitro*, hypersaline stress induces expression of homologues of the Y1074–Y1075 pair (Han *et al.*, 2005), and the *relE*/*parE* toxin family to which Y1075 belongs is known to affect replication and translation (Christensen *et al.*, 2001; Jiang *et al.*, 2002). Some TA systems are involved in both stress response and biofilm development (Wang & Wood, 2011).

The upregulation of TA loci and the reduced expression of metabolic genes by the *phoP* mutant in the flea are reminiscent of the bacterial persistence phenotype – a state of metabolic and replicative dormancy that enables cells to survive hostile environmental conditions. The TA modules are known to inhibit essential energy-requiring cellular processes, such as replication and translation, leading to cell death, bacteriostasis or the formation of dormant persister cells (Buts *et al.*, 2005; Gerdes *et al.*, 2005). Dormant persister cells occur in chronic biofilm-forming bacterial infections, e.g. in *E. coli* urinary tract infections and *Pseudomonas aeruginosa* infection in cystic fibrosis patients where they are metabolically inactive and tolerant to antibiotics, thereby enhancing their recalcitrance to therapeutic treatment (Lewis, 2010). The ability to adopt dormancy as a survival mechanism is not uncommon in bacteria. *Mycobacterium tuberculosis* can persist for years in humans and contains a notable 88 TA loci in its genome (Gerdes & Maisonneuve, 2012; Ramage *et al.*, 2009). These TA systems are implicated in sustaining latency mechanisms in tuberculosis as they relate to low nutritional and hypoxic conditions in granuloma formation (Albrethsen *et al.*, 2013).

*Y. pestis* has been documented to survive in a living flea for up to 396 days, and fleas have been proposed to be ecologically important reservoir hosts that help to preserve the disease cycle from year to year and during quiescent inter-epizootic periods (Burroughs, 1947; Wimsatt & Biggins, 2009). The transcriptional profile of *Y. pestis phoP* mutant growing in the flea exhibits molecular signatures that portend a less metabolically active physiological state. Any semblance of a lower metabolic physiological survival state has not previously been uncovered for *Y. pestis,* but such a survival state could be important for ecological maintenance. The results with the *phoP* mutant suggest that WT *Y. pestis* might also be capable of entering a type of metabolically less active bacteriostatic phase during extended, chronic flea infection or in the soil – representing an alternate persistent reservoir stage in the plague transmission cycle (Wimsatt & Biggins, 2009).

A major consequence of PhoPQ induction is the remodelling of the bacterial surface to confer resistance to outer membrane disrupting agents. Notably, the biofilm phenotype is affected by surface characteristics, such as cell surface charge, LPS structure and the production of adhesins. Several genes that affect the bacterial surface were differentially regulated in the flea. The PhoP-regulated *pgbP*–*pmr* locus and the *ugd* gene, which act to increase surface charge by attaching aminoarabinose to lipid A, were significantly downregulated in the *phoP* mutant. However, *Y. pestis* mutants unable to modify lipid A with aminoarabinose due to deletion of *pbgP* and *ugd* survive and produce normal biofilm-dependent blockage in fleas (Rebeil *et al.*, 2013). Cumulative PhoP-dependent alterations in LPS and the bacterial outer surface may account for the structurally atypical biofilm formation of the *phoP* mutant. For example, the *psa* genes, which encode the pH 6 antigen fimbriae, were strongly derepressed in the *phoP* mutant in the flea. These fimbriae, normally produced only at 37 °C in mammals, might disrupt the dense, cohesive biofilm phenotype, either directly or by masking another adhesin, and contribute to the fragile biofilm phenotype of the *phoP* mutant in the flea. The PhoP mutant's distinct fragmented biofilm phenotype and deficient blocking ability in the flea compared with WT *Y. pestis* might also be the cumulative effect of the large number of differentially expressed genes not directly regulated by PhoP. However, the *phoP* mutant produces less adherent biofilms both *in vitro* and in the flea (Rebeil *et al.*, 2013; Sun *et al.*, 2009).

The transcriptional responses in the *phoP* mutant provide a snapshot of the likely stresses encountered by *Y. pestis* in the flea gut and define the flea gut as a physiologically challenging environment. The results complement the recently published study that details the phenotype of the PhoP mutant during flea infection and its role in producing a transmissible infection (Rebeil *et al.*, 2013). Here, we were able to show that PhoP induction plays an important role in protection against environmental stresses in the flea digestive tract. The more adherent biofilm phenotype that accompanies this response enhances transmissibility. PhoPQ induction in the flea also pre-adapts *Y. pestis* to resist the mammalian innate immune response after transmission (Vadyvaloo *et al.*, 2010). We have also been able to partially characterize a group of genes that function in acid stress. Finally, the indication here that *Y. pestis* has the potential to survive in a less metabolically active physiological state in the flea due to expression of TA systems suggests a persistence mechanism that could help sustain inter-epizootic plague.
